# Conservatism

**Published:** 1905-02

**Authors:** 


### CONSERVATISM.

In reviewing the surgical literature of the past two years one can not fail to note the element of conservatism which prevails in nearly all the departments and specialties. In diseases of the ear and throat several of the once popular operations are now less often advocated. Operations for the correction of uterine displacements are reserved for selected cases and are not employed indiscriminately as heretofore, and in the removal of the uterus and adnexa an attempt is being made to preserve all organs and tissues not actually diseased. Even the much maligned pessary is now recognized as a useful instrument and is being employed with considerable success. The same degree of conservatism is noticeable in many other branches of medicine, and the results attained have amply justified the means employed. No doubt enthusiasm and the example of the ultra progressive surgeon have carried us in the past to extremes, yet this has not proven an unmixed evil, as it has enabled the careful observer to correctly estimate the comparative advantages of the various methods.—*New England Med. Journal.*

